# 

**Published:** 1906-03

**Authors:** 


### THE AMERICAN
### Journal of Dental Science

**Founded, Owned, Edited and Published by the Dental Profession of America.**

**THIRD SERIES.**

VOLUME XXXVII. MARCH, 1906. NO. 3.

EDITORS:
WM. GIRD BEECROFT, D. D. S., Madison, Wis. B. H. TEAGUE, D. D. S., Aiken, S. C
Published on the 10th day of each month
Subscription Price, $1 00 per year Foreign countries, $1.50 per year.
*Entered as Second-Class Matter, Madison, Wis.*
Address all communications, both business and editorial, to
**The Dental Publishing Company, Madison, Wisconsin.**

### SOCIETY ANNOUNCEMENTS.

Iowa State Dental Society meets at Des Moines, May 1st, 2nd and 3rd inclusive.

Missouri State Dental Society meets at Springfield, June 5th, 6th and 7th inclusive. All ethical dentists invited to attend.

Kentucky State Dental Association meets at Dawson Springs, June 4th, 5th and 6th. A cordial invitation is extended to the profession to be present.

Florida State Dental Society meets at the Continental Hotel, Atlantic Beach (near Jacksonville), June 15th continuing three days. All ethical dentists urged to be present.

Alabama State Dental Association meets in the Commercial Club rooms, Mobile, May 8th to the 10th. All ethical dentists urged to attend. One and one-third fare on all railroads on the certificate plan.

The Board of Trustees of Barnes University of St. Louis, announces the following as the present officers of the Dental Department: Dr. Geo. H. Owen, Dean; Otto J. Fruth, S. H. Voyles, C. O. Simpson, W. B. Arthur, T. G. Donnell, W. H. Love, C. P. Strawn.

The new building of the Dental Department will be completed about June 1st, 1906, and will be complete in all its appointments.

